# Assessment of urinary 6‐oxo‐pipecolic acid as a biomarker for ALDH7A1 deficiency

**DOI:** 10.1002/jimd.12783

**Published:** 2024-07-22

**Authors:** Youssef Khalil, Emma Footitt, Reddy Vootukuri, Michael F. Wempe, Curtis R. Coughlin, Spyros Batzios, Matthew P. Wilson, Viktor Kožich, Peter T. Clayton, Philippa B. Mills

**Affiliations:** ^1^ Genetics and Genomic Medicine University College London Great Ormond Street Institute of Child Health London UK; ^2^ Department of Metabolic Paediatrics Great Ormond Street Hospital London UK; ^3^ School of Pharmacy, Department of Pharmaceutical Sciences University of Colorado Aurora Colorado USA; ^4^ Department of Pediatrics, Section of Clinical Genetics and Metabolism University of Colorado School of Medicine Aurora Colorado USA; ^5^ Laboratory for Molecular Diagnosis Center for Human Genetics, KU Leuven Leuven Belgium; ^6^ Department of Pediatrics and Inherited Metabolic Disorders Charles University‐First Faculty of Medicine and General University Hospital in Prague Prague Czech Republic

**Keywords:** 6‐oxo‐pipecolic acid, ALDH7A1 deficiency, aminoadipic semialdehyde, piperideine‐6‐carboxylate, pyridoxine‐dependent epilepsy

## Abstract

ALDH7A1 deficiency is an epileptic encephalopathy whose seizures respond to treatment with supraphysiological doses of pyridoxine. It arises as a result of damaging variants in *ALDH7A1,* a gene in the lysine catabolism pathway. α‐Aminoadipic semialdehyde (α‐AASA) and Δ^1^‐piperideine‐6‐carboxylate (P6C), which accumulate because of the block in the lysine pathway, are diagnostic biomarkers for this disorder. Recently, it has been reported that 6‐oxo‐pipecolic acid (6‐oxo‐PIP) also accumulates in the urine, CSF and plasma of ALDH7A1‐deficient individuals and that, given its improved stability, it may be a more suitable biomarker for this disorder. This study measured 6‐oxo‐PIP in urine from a cohort of 30 patients where α‐AASA was elevated and showed that it was above the normal range in all those above 6 months of age. However, 6‐oxo‐PIP levels were within the normal range in 33% of the patients below 6 months of age. Levels increased with age and correlated with a decrease in α‐AASA levels. Longitudinal analysis of urine samples from ALDH7A1‐deficient patients who were on a lysine restricted diet whilst receiving supraphysiological doses of pyridoxine showed that levels of 6‐oxo‐PIP remained elevated whilst α‐AASA decreased. Similar to α‐AASA, we found that elevated urinary excretion of 6‐oxo‐PIP can also occur in individuals with molybdenum cofactor deficiency. This study demonstrates that urinary 6‐oxo‐PIP may not be a suitable biomarker for ALDH7A1 deficiency in neonates. However, further studies are needed to understand the biochemistry leading to its accumulation and its potential long‐term side effects.

## 1 INTRODUCTION

**FIGURE 1:**
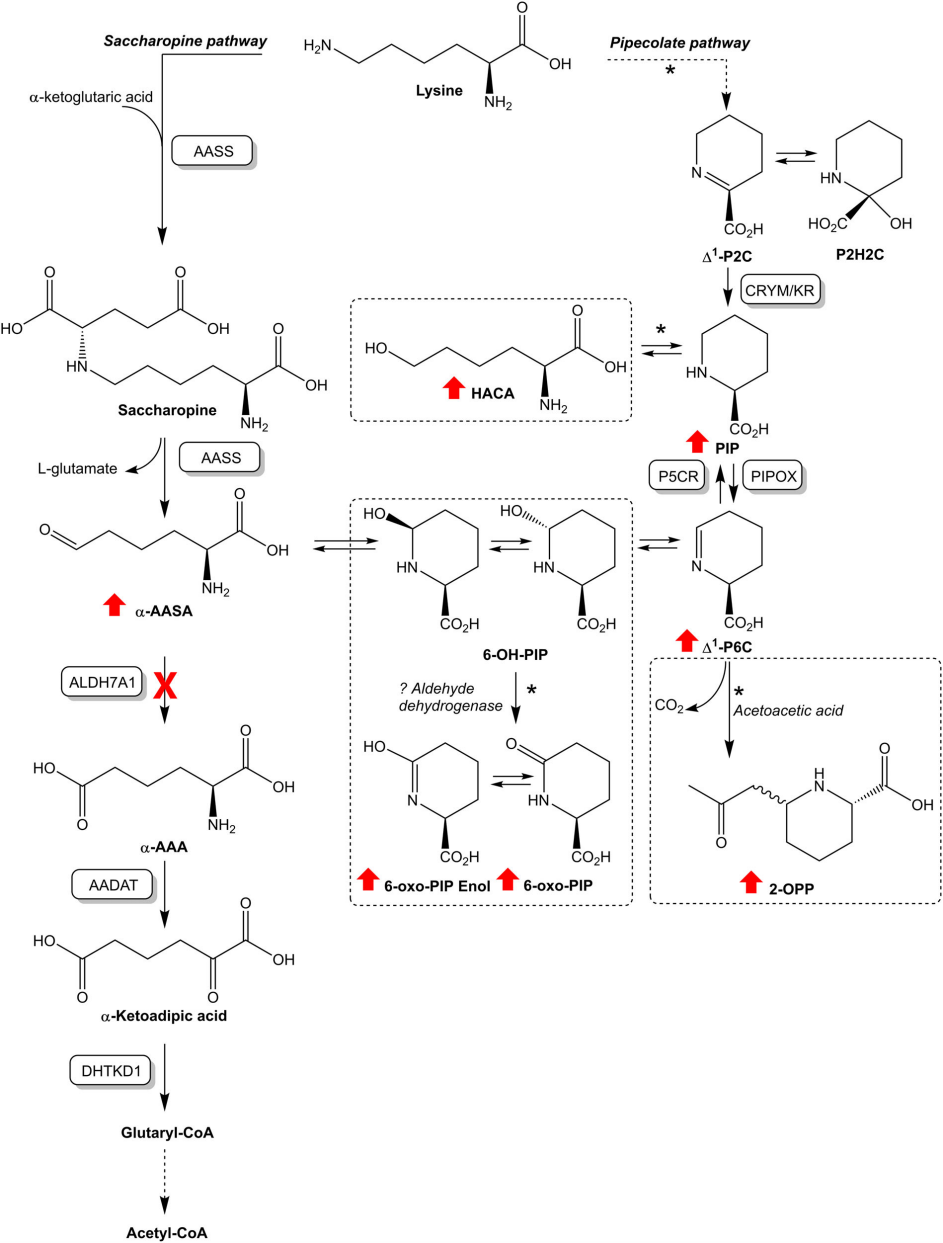
The lysine oxidation pathway. Biomarkers of ALDH7A1 deficiency are highlighted with a red arrow. 6‐oxo‐PIP and its enol form are formed by the oxidation of 6‐OH‐PIP, an α‐AASA and Δ^1^‐P6C intermediate. PIP, pipecolate; Δ^1^‐P6C, Δ^1^‐piperideine‐6‐carboxylate; 6‐OH‐PIP, 6‐hydroxy‐pipecolate; 2‐OPP, 2*S*,6*S*‐/2*S*,6*R*‐oxopropylpiperidine‐2‐carboxylic acid; 6‐oxo‐PIP, 6‐oxo‐pipecolate; Δ^1^‐P2C, Δ^1^‐piperideine‐2‐carboxylate; P2H2C, piperidine‐2‐hydroxy‐2‐carboxylate; HACA, 6‐hydroxy‐2‐aminocaproic acid; α‐AASA, aminoadipic semialdehyde; AAA, aminoadipic acid; AASS, aminoadipic semialdehyde synthase; AADAT, aminoadipate aminotransferase; CRYM/KR, μ‐crystallin/ketimine reductase; DHTKD1, 2‐oxoadipate dehydrogenase complex component E1; P5CR, pyrroline‐5‐carboxylate reductase 1; PIPOX, pipecolic acid and sarcosine oxidase. Asterisk indicates a speculative step. Dashed arrow indicates a multi‐enzyme step.

ALDH7A1 deficiency is an autosomal recessive epileptic encephalopathy that classically presents within the first weeks to months of life. This disorder does not usually respond to treatment with general anticonvulsants but responds to pyridoxine (a form of vitamin B_6_) supplementation. Pathogenic variants in *ALDH7A1*, which encodes α‐aminoadipic semialdehyde (α‐AASA) dehydrogenase in the lysine catabolism pathway, result in accumulation of α‐AASA and its cyclic form, Δ^1^‐piperideine‐6‐carboxylate (P6C), with which it is in equilibrium (Figure 1).^1^ P6C complexes with pyridoxal 5′‐phosphate (PLP), the only B_6_ vitamer that acts as an enzyme cofactor, via Knoevenagel condensation resulting in its inactivation.^1^ This results in the depletion of bioavailable PLP required for the various reactions for which it acts as a cofactor, many of which are involved in neurotransmitter metabolism, and results in an epilepsy phenotype. Despite the response of seizures to pyridoxine treatment, long‐term neurocognitive deficit occurs in patients with up to 75% suffering from some level of developmental delay, often irrespective of early treatment.^2^ Alongside pyridoxine supplementation, lysine‐restricted and arginine supplemented diets, to reduce the neurotoxic levels of α‐AASA/P6C, have however shown some promise.^3^ ALDH7A1 can also be inhibited by accumulating sulphite in sulphite oxidase (SUOX) or molybdenum cofactor deficiency (MoCD) thereby eliciting a secondary ALDH7A1 deficiency.^4^, ^5^, ^6^

Current biochemical diagnosis of ALDH7A1 deficiency relies on the measurement of α‐AASA and P6C, upstream metabolites which are elevated in urine, plasma, dried blood spots and CSF.^1^, ^7^, ^8^, ^9^ However, α‐AASA/P6C are unstable at room temperature with up to 50% urinary α‐AASA degradation reported within 24 h.^7^, ^10^ Urine samples must therefore be stored at −80°C before analysis to minimise the incidence of false negative results and allow α‐AASA/P6C to be used as biomarkers for treatment monitoring. α‐AASA in dried blood spots has been shown to have better stability at room temperature for up to 3 days although up to 70% degradation can occur within 2 weeks.^9^ Analysis of urinary α‐AASA/P6C remains the preferred diagnostic option.

Recently, three novel ALDH7A1 deficiency biomarkers have been reported, 6‐oxo‐pipecolic acid (6‐oxo‐PIP, also known as 6‐oxopiperidine‐2‐carboxylic acid),^11^, ^12^ 2*S*,6*S‐/*2*S*,6*R*‐oxopropylpiperidine‐2‐carboxylic acid (2‐OPP),^13^ and 6‐hydroxy‐2‐aminocaproic acid (HACA).^14^ 2‐OPP and 6‐oxo‐PIP have been shown to be elevated in urine, plasma and CSF of ALDH7A1‐deficient patients, while HACA has only been tested and detected in plasma. Importantly, 2‐OPP and 6‐oxo‐PIP are stable for months at room temperature, rendering them potentially suitable biomarkers for newborn screening. The formation of 6‐oxo‐PIP derives from 6‐OH‐pipecolic acid, the intermediate between P6C and α‐AASA, which undergoes oxidation of its secondary alcohol by an unidentified aldehyde dehydrogenase (Figure 1).^12^ 2‐OPP is formed by the reaction of P6C with the ketone body acetoacetate, in which its level of formation is dependent on the patient's ketotic state.^13^ HACA is suggested to be in an enzyme‐independent equilibrium with pipecolic acid.^14^

In this study, we measured urinary 6‐oxo‐PIP in a cohort of ALDH7A1‐deficient patients for whom the diagnosis had been genetically and biochemically confirmed and for patients in whom ALDH7A1 deficiency had been diagnosed biochemically, that is, had elevated urinary α‐AASA, but for whom we do not have any further details. The levels of 6‐oxo‐PIP in these two groups were compared to those seen in patients with seizures of unknown origin who had levels of α‐AASA within the control range. Given that it is well established that urinary α‐AASA can also be raised in MoCD and SUOX deficiency (SOXD) patients, we also measured 6‐oxo‐PIP in a cohort of genetically‐defined patients with these two disorders. This allowed us to determine the viability of 6‐oxo‐PIP as a biomarker for ALDH7A1 deficiency screening. Urinary 6‐oxo‐PIP and α‐AASA levels were also quantified in longitudinal samples from genetically defined ALDH7A1‐deficient patients receiving pyridoxine supplementation and lysine restriction.

## 2 METHODS

### 2.1 Materials

6‐oxo‐pipecolic acid and 9‐fluorenylmethoxycarbonyl chloride (FMOC‐Chloride) were purchased from Sigma‐Aldrich. D,L‐2‐Amino‐1,6‐hexanedioic‐2,5,5‐d_3_ (D_3_‐AAA) and creatinine‐d_3_ were purchased from CDN Isotopes. All other reagents were of analytical grade. D_3_‐6‐oxoPIP was synthesised as described previously.^12^

### 2.2 Patient samples

Urine samples (*n* = 75) were available from 30 patients which had elevated urinary α‐AASA concentrations due to a primary or secondary ALDH7A1 deficiency. This included samples from 12 patients who were genetically confirmed to have biallelic *ALDH7A1* variants. The other samples were anonymised and hence we had no genetic information. Control urine samples (*n* = 80) were from patients with seizures of unknown origin with normal urine α‐AASA levels. Samples had been sent to our laboratory for the measurement of α‐AASA for diagnostic or monitoring purposes. It is possible that some of these samples were from patients that were being treated with pyridoxine. Urine samples were also collected from five MoCD patients (MoCD‐A, *n* = 1; MoCD‐B, *n* = 4) and two SOXD patients whose diagnosis had been genetically confirmed.

### 2.3 Liquid chromatography–mass spectrometry

Samples for α‐AASA analysis were prepared as follows. 1 mL of urine was centrifuged at 16 000 × *g* to remove any particulate matter prior to 10 μL of the sample being mixed with 10 μL 0.1 mmol/L d_3_‐AAA internal standard. Deionised H_2_O (40 μL) and 1 mM borate buffer pH 10.4 (125 μL) were added to the urine/internal standard mix, and derivatised by addition of 125 μL of 5.8 mM FMOC‐Chloride. Urinary α‐AASA was measured by LC–MS/MS on a Waters Alliance 2695 LC coupled to a Waters Micromass Quattro Micro. Ten μL of the reaction mixture was analysed on a Discovery® HS F5 HPLC column (5 cm × 2.1 mm, 5 μm) using mobile phase A (4 mM ammonium acetate, pH 5) and B (100% acetonitrile) with a linear gradient as described previously.^15^ α‐AASA and d_3_‐AAA were detected by multiple reaction monitoring (MRM) in negative ion mode using the parameters in Table S1. The α‐AASA was semi‐quantified based on an α‐AASA:d_3_‐AAA (1:1) ratio. Urine creatinine levels were measured by LC–MS/MS using established methods.^15^

Samples for 6‐oxo‐PIP analysis were prepared by mixing 20 μL of urine with 20 μL of 10 μmol/L d_3_‐6‐oxo‐PIP internal standard. The mix was then diluted with 80 μL 1:1 methanol: acetonitrile. A 10 point 6‐oxo‐PIP standard curve was generated with concentrations from 1 to 500 μmol/L. Urine 6‐oxo‐PIP was measured by LC–MS/MS on a Waters Acquity UPLC coupled to an electrospray Xevo TQ‐S triple quadrupole mass spectrometer. Ten microliters of the reaction mixture were analysed on an ACQUITY UPLC® HSS T3 (1.8 μm × 2.1 mm × 15 mm) using mobile phase A (10 mM ammonium acetate, 0.1% formic acid) and B (1:1 methanol: acetonitrile), at a flow rate of 0.4 mL/min. A linear gradient was used: 0–2.5 min 99% A, 2.5–4.5 min 95% B held until 5.1 min, then 5.1–8 min 99% A. 6‐oxo‐PIP and d_3_‐6‐oxo‐PIP were detected by MRM in positive ion mode using the parameters in Table S1.

## 3 RESULTS

### 3.1 6‐oxo‐PIP age‐related control ranges

**FIGURE 2:**
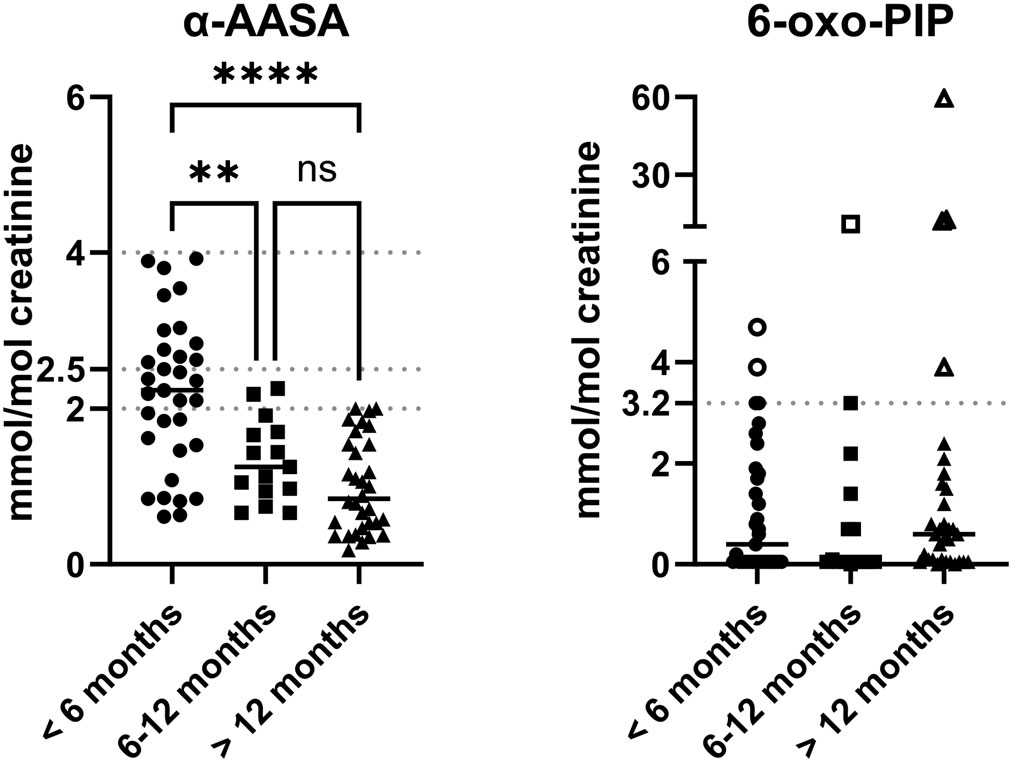
Urine α‐AASA and 6‐oxo‐PIP control ranges. Urine was from a cohort of 80 individuals with seizures of unknown origin but with α‐AASA concentrations within the normal range. Samples were grouped based on established age‐related urine α‐AASA control ranges (mmol/mol creatinine): <4, under 6 months; <2.5, 6–12 months; <2, above 12 months of age. Urine α‐AASA levels were significantly higher in individuals under 6 months of age. Urine 6‐oxo‐PIP control levels were not significantly different based on age. Thus, a control range was established based on the 97.5th centile (0–3.2 mmol/mol creatinine). Seven control samples with 6‐oxo‐PIP levels above the control range were determined as outliers (open symbols). *P*‐values: **<0.01; ****<0.0001; ns, not significant.

Control ranges for urinary 6‐oxo‐PIP were established using samples from patients with seizures of unknown origin, which had been shown to have α‐AASA concentrations within the control range. Samples were grouped based on ages similar to those published for urinary α‐AASA analysis (<6 months, 6–12 months and >12 months)^7^, ^15^ (Figure 2). Individuals under 6 months of age (*n* = 33) had significantly higher α‐AASA levels compared to those above 6 months of age (*n* = 47) which agrees with previously reported age‐related findings.^7^, ^15^ In contrast, there were no significant differences in the 6‐oxo‐PIP levels between those 3 age groups. As a result, the control range was determined from the cohort irrespective of age and 7 outliers were found using the ROUT method (*Q* = 0.5%). After exclusion of the outliers from the data set, the 6‐oxo‐PIP median, 2.5th and 97.5th centile were 0.2, 0 and 3.2 mmol/mol creatinine, respectively. Thus, a urine 6‐oxo‐PIP control range of 0–3.2 mmol/mol creatinine was established. The 6‐oxo‐PIP levels of the 7 outliers ranged from 3.9 to 59.4 mmol/mol creatinine.

### 3.2 Urinary α‐AASA and 6‐oxo‐PIP concentrations in ALDH7A1 deficiency

**FIGURE 3:**
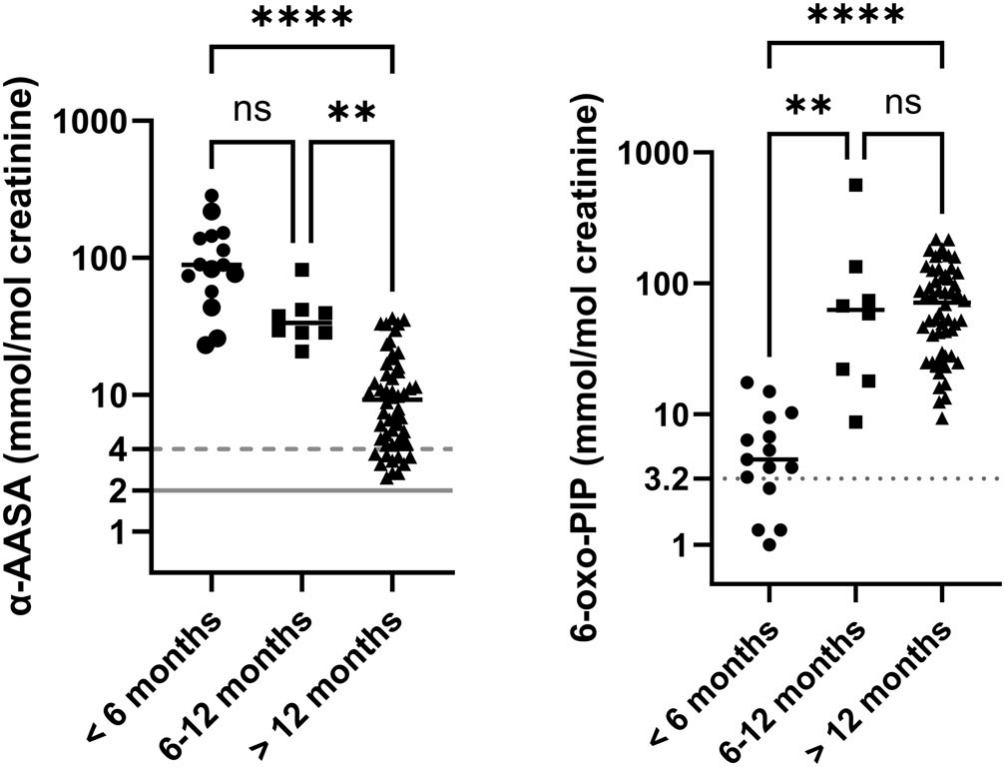
6‐oxo‐PIP levels in urine samples shown to have elevated α‐AASA levels. Dotted line, 6‐oxo‐PIP upper control limit; dashed line, α‐AASA upper control limit below 6 months of age; solid line, α‐AASA upper control limit above 12 months of age; black bar, median. α‐AASA control range for 6–12 months of age = 2.5 mmol/mol creatinine. *P*‐values: **<0.01; ****<0.0001.

**FIGURE 4:**
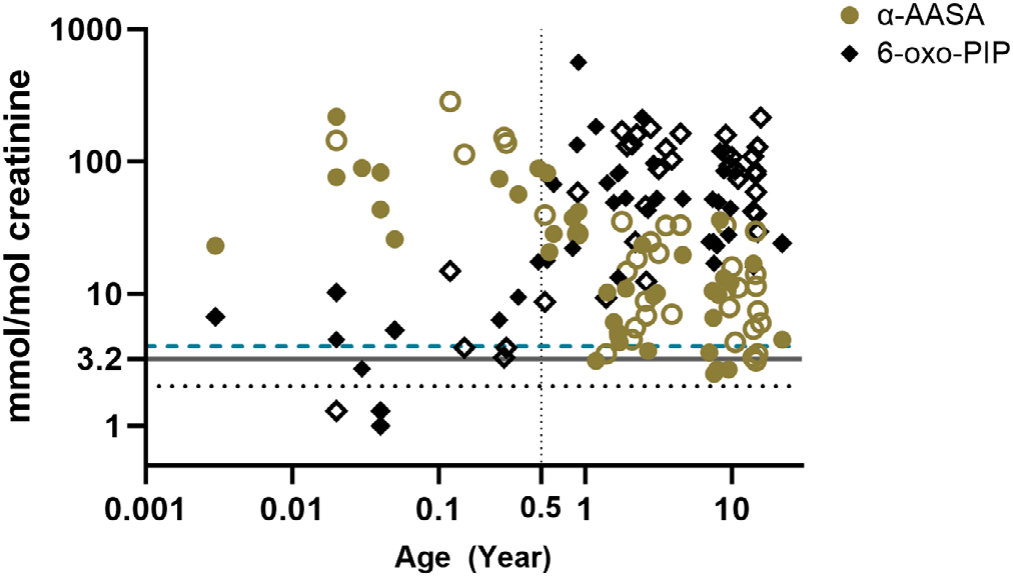
Age‐related changes of 6‐oxo‐PIP concentrations in samples from patients with elevated α‐AASA concentrations. Open symbols, patients confirmed as having biallelic *ALDH7A1* variants. Dashed line, α‐AASA control range <6 months old; dotted line, α‐AASA control range >12 months old; solid line, 6‐oxo‐PIP upper limit of control range for all age groups. The genetically confirmed patients will have been on pyridoxine supplementation and in some instances lysine restriction while for the others the treatment regime is unknown.

A total of 75 samples were analysed from 30 individuals that had been identified biochemically as ALDH7A1‐deficient on the basis of an elevated α‐AASA level. Urine 6‐oxo‐PIP levels were elevated in all patients older than 6 months old, while 4 out of 15 samples from patients under 6 months old, one of whom has been genetically confirmed, had levels within the normal range. We do not have genetic details for the other three individuals as the samples were anonymised prior to analysis. Moreover, three patients below 4 months old had ‘borderline’ 6‐oxo‐PIP levels, that is, 3.30, 3.85 and 3.87 mmol/mol creatinine whilst their α‐AASA levels were between 113 and 152 mmol/mol creatinine (Figure 3). The mean concentration of urinary 6‐oxo‐PIP in patients under 6 months old was 5.5 ± 5.4 mmol/mol creatinine in those genetically and biochemically defined ALDH7A1‐deficient patients, and 6.5 ± 4.9 mmol/mol creatinine in those only biochemically defined, compared to α‐AASA means of 166.9 ± 67.5 and 77.8 ± 55.2 mmol/mol creatinine, respectively (Table 1). The 6‐oxo‐PIP mean and median concentrations were significantly higher in patients above 6 months of age (*p* < 0.01), whereas that of α‐AASA were significantly lower in patients above 12 months of age (*p* < 0.0001). Comparison of α‐AASA and 6‐oxo‐PIP levels relative to the age when the sample was collected revealed that while levels of 6‐oxo‐PIP increase with age, levels of α‐AASA decrease (Figure 4). A two‐tailed spearman test indicates a significant, but weak negative correlation between urinary α‐AASA and 6‐oxo‐PIP in these patients (*p* < 0.05; Spearman *r* = −0.28). This correlation is not significant however, when only the genetically and biochemically defined cohort is compared. Two genetically confirmed ALDH7A1‐deficient patients above the age of 12 years had normal α‐AASA levels while their 6‐oxo‐PIP levels were ~20–28 mmol/mol creatinine (Figure S1). Both of these individuals are on a lysine restricted diet.

**TABLE 1:** Urine α‐AASA and 6‐oxo‐PIP levels in ALDH7A1‐deficient patients based on age group. Individuals were identified biochemically as ALDH7A1‐deficient on the basis of an elevated α‐AASA level. CR, control range; the values are stated in the table.

	α‐AASA			6‐oxo‐PIP		
Age (months)	<6	6–12	>12	<6	6–12	>12
Control range (CR) (mmol/mol creatinine)	<4	<2.5	<2	<3.2	<3.2	<3.2
*Genetically and biochemically defined ALDH7A1‐deficient patients*						
Mean (x̄)	167.6		13.6	5.5		99.0
Standard deviation	67.5		10.6	5.4		54.2
Median (*M*)	144.2		9.7	3.9		98.1
Min–max	113.8–284.5	28.4–39.5	3.1–35.2	1.3–14.9	8.7–58.2	9.3–215.1
Factor change (x̄/CR; *M*/CR)	41.9; 36.1		6.8; 4.9	1.7; 1.2		30.9; 30.6
Number of patients (*n*)	5	2	28	5	2	28
*Biochemically defined ALDH7A1‐deficient patients*						
Mean (x̄)	77.8	39.9	9.7	6.5	146.7	62.4
Standard deviation	55.2	21.8	7.9	4.9	209.2	50.9
Median (*M*)	75.1	33.5	8.1	5.8	70.6	50.5
Min–max	23–218.5	20.7–81.6	2.47–36.1	1–17.4	17.9–564.9	13.3–216.8
Factor change (x̄/CR; *M*/CR)	19.5; 18.8	16.0; 13.4	4.9; 4.1	2.0; 1.8	45.8; 22.1	19.5; 15.8
Number of patients (*n*)	10	6	24	10	6	24

### 3.3 Longitudinal analysis of urinary α‐AASA and 6‐oxo‐PIP concentrations in ALDH7A1‐deficient patients

**FIGURE 5:**
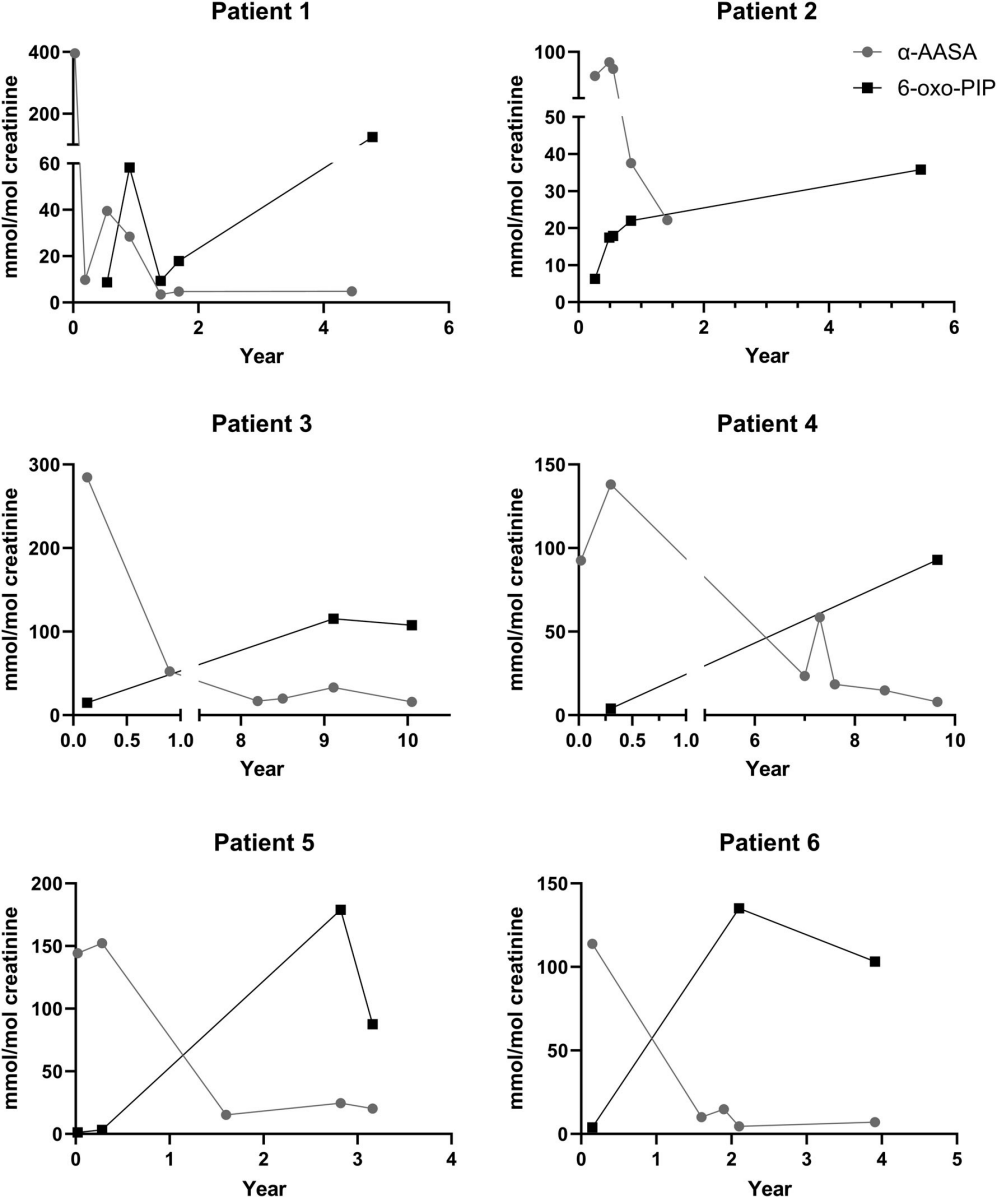
Longitudinal analysis of urinary α‐AASA and 6‐oxo‐PIP for ALDH7A1‐deficient patients. The diagnosis of all of these patients was confirmed genetically. All patients had been on pyridoxine supplementation and a lysine restricted diet as described in Table 2.

Urine α‐AASA and 6‐oxo‐PIP levels were quantified in longitudinal samples from six genetically confirmed ALDH7A1‐deficient patients. Their treatment regimen is detailed in Table 2. An increase was seen in urinary 6‐oxo‐PIP with increasing age, whilst α‐AASA decreased (Figure 5).

**TABLE 2:** Treatment regimen for ALDH7A1‐deficient patients 1–6.

Patient	Initial 6‐oxo‐PIP level^a^ (age – years)	Pyridoxine treatment start age (days)	Lysine restriction start age (years)	Adherence to diet
1	8.7 (0.54)	10	0.11	Full
2	6.3 (0.26)	9	0.51	Full
3	14.9 (0.13)	14	8.17	Partial
4	3.9 (0.30)	9	6.87	Partial
5	1.3^b^ (0.02)	10	0.28	Partial
6	3.9 (0.15)	1	0.44	Full

^a^ mmol/mol creatinine.

^b^ Within control range (<3.2 mmol/mol creatinine).

### 3.4 Specificity of 6‐oxo‐PIP for ALDH7A1 deficiency

**FIGURE 6:**
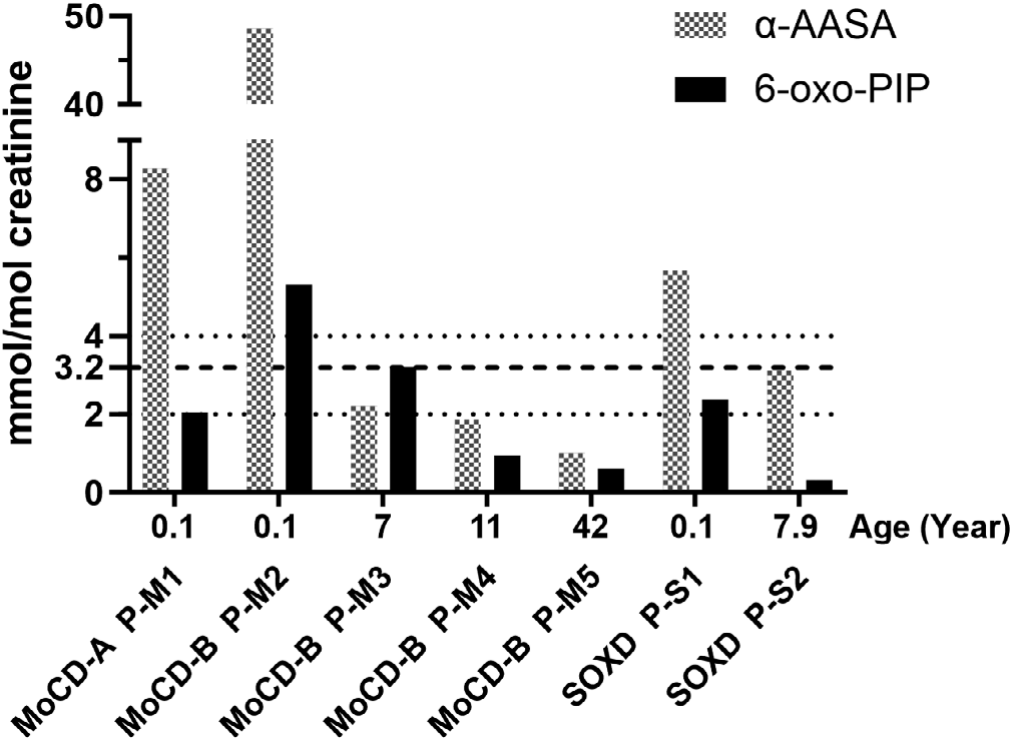
Urine α‐AASA and 6‐oxo‐PIP levels in MoCD‐A/B and SOXD patients. Dotted lines, α‐AASA control upper limits for <6 and >12 months old (4 and 2 mmol/mol creatinine, respectively); Dashed line, 6‐oxo‐PIP control upper limit.

We have shown previously that concentrations of α‐AASA may also be elevated in patients with MoCD and SOXD.^4^ Hence, we measured 6‐oxo‐PIP in a cohort of individuals with these disorders whose α‐AASA levels had been reported previously^6^ (*n* = 1; MoCD‐A, *n* = 4; MoCD‐B and *n* = 2, SOXD). This was a separate cohort of samples to that described above for which α‐AASA had been shown to be elevated (*n* = 75). The urine α‐AASA concentration was elevated in 2 of the MoCD‐B patients (P‐M1 and P‐M2) and moderately elevated in one SOXD patient (P‐S1) (Figure 6).^6^ 6‐oxo‐PIP urine levels were only above the normal range in one of these three patients, that is, P‐M2 (MoCD‐B). Patient P‐M3 (MoCD‐B), who was older than 12 months of age and had borderline α‐AASA excretion, also had borderline levels of 6‐oxo‐PIP.

## 4 DISCUSSION

Since the identification of 6‐oxo‐PIP as a potential biomarker for ALDH7A1 deficiency, a limited number of studies have been conducted to validate these findings.^11^, ^13^ They report elevated urinary 6‐oxo‐PIP in all their patients in addition to its elevation in plasma and CSF. However, there is no clear indication on how 6‐oxo‐PIP excretion correlates with α‐AASA accumulation, age, and treatment. Herein, we demonstrate that 6‐oxo‐PIP is significantly elevated in urine samples of a cohort of 30 patients who have elevated α‐AASA levels. However, whether excretion of 6‐oxo‐PIP is elevated can depend on the age of the patient at time of testing with some individuals below the age of 6 months having levels within the normal range even though the concentration of α‐AASA in their urine is elevated. The study of longitudinal samples from 6 patients on treatment shows continuous increase in urinary excretion of 6‐oxo‐PIP with age while α‐AASA levels, in general, decrease.

Urinary 6‐oxo‐PIP excretion was elevated in seven control samples, that is, outliers, where the urine α‐AASA level was within the age‐related reference range. There are several variables that might contribute to the level of 6‐oxo‐PIP excretion seen in these individuals. The first is diet and gut flora. 6‐oxo‐PIP is the cyclised lactam of α‐AAA. Cyclisation can occur due to heat with a 6‐oxo‐PIP:AAA equilibrium (3:1) being reached after 2 h at 100°C.^16^ α‐AAA is present in dairy and protein rich foods,^17^ thus there is potential for diet to influence 6‐oxo‐PIP urinary excretion. 6‐Oxo‐PIP has also been elevated in the plasma of children with raised cholesterol who were fed navy beans.^18^ Gut flora may also contribute to an elevated concentration. Fungi comprise 0.1% of the gut flora and can produce aminoadipic acid and 6‐oxo‐PIP as has been established in *Penicillium chrysogenum* during the synthesis of Penicillin V.^19^, ^20^ The second potential contributing variable is the medication patients are taking. Anti‐epileptic drugs and antibiotics may potentially lead to elevated 6‐oxo‐PIP levels, specifically the anti‐epileptic drug Vigabatrin,^21^ and the antibiotic Flucloxacillin,^22^ as can chronic acetaminophen treatment.^21^ These cause reduced activity of 5‐oxoprolinase, and therefore conversion of 5‐oxoproline to glutamate, due to disruption of the gamma‐glutamyl cycle. Whilst this results primarily in pyroglutamic aciduria and elevated 5‐oxoproline excretion, 5‐oxo‐prolinase can also, to a lesser extent, catalyse the conversion of 6‐oxo‐PIP to aminoadipic acid.^23^ One could therefore hypothesise that reduced activity of 5‐oxoprolinase could result in increased 6‐oxo‐PIP excretion. 5‐oxo‐proline excretion was not elevated in one control sample we analysed where 6‐oxo‐PIP excretion was elevated (data not shown). The final variable we must also consider is that the control samples are from patients with seizures and whilst they do not have elevated levels of α‐AASA, and therefore are not ALDH7A1‐deficient, it is possible that other underlying causes are leading to elevated 6‐oxo‐PIP excretion. 6‐oxo‐PIP has been found to be elevated in a patient with α‐ketoadipic/α‐aminoadipic aciduria caused by variants in the *DHTKD1* gene. This gene encodes a subunit of a mitochondrial 2‐oxoglutarate‐dehydrogenase‐complex‐like protein that catalyses the conversion of 2‐oxoadipate to glutaryl‐CoA in the lysine degradation pathway. The phenotype of individuals with *DHTKD1* gene variants ranges from asymptomatic to severe intellectual disability, muscular hypotonia, developmental delay, ataxia, and epilepsy.^24^ Notably, *DHTKD1* is also associated with type 2 diabetes,^25^ and it has been reported that 6‐oxo‐PIP is significantly elevated in adult type 2 diabetes patients.^26^

Our findings show that in one genetically confirmed patient under 6 months of age, 6‐oxo‐PIP was within the control range. Similarly, three patients with high α‐AASA levels for whom we have no genetic details, had normal 6‐oxo‐PIP levels. A further three genetically confirmed patients between the age of 2 and 4 months showed borderline 6‐oxo‐PIP levels (3.3, 3.85 and 3.87; control range 0–3.2 μmol/mmol creatinine) while their α‐AASA levels were 113–152 mmol/mol creatinine. Based on these findings, 7 out of 15 samples for patients under 6 months old would have shown a false negative or inconclusive result for ALDH7A1 deficiency if biochemical diagnosis had been dependent on urinary 6‐oxo‐PIP levels solely. In older patients where α‐AASA excretion has normalised, 6‐oxo‐PIP remained elevated (Figure S1).

In our cohort, two MoCD deficiency patients (P‐M1 and P‐M2) and one SUOX deficiency patient (P‐S1) had elevated α‐AASA. SUOX is an enzyme involved in the catabolism of cysteine where it oxidises sulphite to sulfate. SUOX deficiency leads to accumulation of sulphite and S‐sulphocysteine, with the former having an inhibitory effect on ALDH7A1 thus leading to the accumulation of α‐AASA.^4^ In MoCD patients, SUOX is also deficient in addition to a deficiency of xanthine dehydrogenase and aldehyde oxidase. Only P‐M2 with substantially elevated α‐AASA excretion also had elevated 6‐oxo‐PIP excretion, albeit at the lower range in comparison to ALDH7A1‐deficient patients. As our data suggests that 6‐oxo‐PIP excretion is age dependant and increases with age, the 6‐oxo‐PIP levels in this MoCD and SUOX deficient cohort may be due to their age (less than 6 months old). Notably, patients above the age of 6 months showed normal to borderline α‐AASA and normal 6‐oxo‐PIP levels which suggest sufficient ALDH7A1 activity. More importantly, the elevation of 6‐oxo‐PIP in one of the five MoCD patients tested suggests its generation is not specific to primary ALDH7A1 deficiency.

The increase of 6‐oxo‐PIP excretion with age in ALDH7A1‐deficient patients, in contrast to the decrease in α‐AASA, could be attributed to multiple factors that are as yet to be identified. It is hypothesised that 6‐oxo‐PIP is generated as a result of oxidation of 6‐OH‐PIP by an unidentified cytosolic aldehyde dehydrogenase,^12^ and it may be that the expression of this enzyme is age dependent as has been shown with other aldehyde dehydrogenases.^27^ This would however not explain why the levels of 6‐oxo‐PIP do not decrease similarly to α‐AASA when patients are on a lysine restricted diet.

Two other factors that may contribute to the age‐related pattern seen include pyridoxine supplementation and oxidative stress. Current treatment of ALDH7A1‐deficient patients involves life‐long supplementation with supraphysiological doses of pyridoxine. Whilst this prevents the seizures that are part of this disorder from occurring, it does not prevent neurodevelopmental delay in approximately 75% of the patients. This is believed to occur due to an accumulation of α‐AASA/P6C, which have been proposed as being neurotoxic. Hence, a lysine restriction diet and/or arginine supplementation, which aims to inhibit enteral lysine uptake at the level of the transporters,^28^ has been used in many patients to decrease the levels of these neurotoxic metabolites. Yet, little is understood on the long‐term metabolic effects of pyridoxine supplementation. ALDH7A1‐deficient patients treated with high doses of pyridoxine have been reported to have decreased plasma levels of glycine among other amino acids.^29^ A similar profile was seen in *ALDH7A1* knockout neural progenitor cells treated with high doses of pyridoxine. Notably, the levels of oxo‐proline and glycine, two gamma‐glutamyl cycle metabolites, were decreased in patient plasma on pyridoxine and *ALDH7A1* knockout neural progenitor cells treated with pyridoxine compared to controls.^29^ The gamma‐glutamyl cycle is part of the glutathione biosynthesis pathway which partly functions as an antioxidant. Glutathione is mostly present in a reduced state in the cytosol and can scavenge free radicals on its own or as a cofactor to antioxidant enzymes. Thus, the impact of pyridoxine supplementation on the gamma‐glutamyl cycle and glutathione metabolism could lead to a greater susceptibility to free radical damage. In addition, it has been reported that mitochondrial and cytosolic ALDH7A1 have antioxidant properties.^30^ This has been demonstrated in Chinese hamster ovary cells where expression of ALDH7A1 provided protection against lipid peroxidation‐derived aldehydes and hydrogen peroxide.^31^ Further evidence of ALDH7A1 protecting against oxidative stress has been seen in an *ALDH7A1* knockout mouse brain with elevated levels of methionine sulfoxide, an oxidative stress biomarker, present.^32^ How though could the combination of greater susceptibility to free radical damage because of the high dose of pyridoxine supplementation and the reduced protection from free radical damage due to reduced ALDH7A1 activity result in the formation of 6‐oxo‐PIP? Preliminary experiments have shown that 6‐oxo‐PIP can be formed from the incubation of α‐AASA/P6C with hydrogen peroxide at pH 7.6 in the presence of ferrous chloride by Fenton reaction (data not shown). We hypothesise that the hydroxyl radical oxidises the α‐AASA/P6C intermediate 2‐OH‐PIP to form 6‐oxo‐PIP. It is however unknown if such a reaction would occur in vivo. Measuring 6‐oxo‐PIP production after subjecting ALDH7A1‐deficient cells to oxidative stress may shed light on this. As of yet, there has been no report of 6‐oxo‐PIP detection in ALDH7A1‐deficient cell lines. Measuring α‐AASA and 6‐oxo‐PIP concentrations in fibroblasts exposed to high lysine concentrations from two patients with biallelic pathogenic variants in *ALDH7A1* showed an accumulation of α‐AASA as expected, while 6‐oxo‐PIP was undetected (Figure S2). Stable isotope labelled studies on these cell lines are needed to determine whether 6‐oxo‐PIP was not generated due to the lack of sufficient oxidative stress. Alternatively, it may be that the proposed unknown aldehyde dehydrogenase required to oxidise 6‐OH‐PIP to 6‐oxo‐PIP is not expressed in fibroblasts.

Going forward, further investigations will be needed given that our interpretations only consider the age‐dependent levels of 6‐oxo‐PIP in urine and not in blood or CSF. Additional data is needed to compare the biomarker profile in these different sample types based on age and treatment to determine the viability of 6‐oxo‐PIP, particularly in dried blood spots, as a biomarker for newborn screening.

## 5 CONCLUSION

The increase in accumulation of 6‐oxo‐PIP with age raises further questions on the biochemistry of the pathophysiology of ALDH7A1 deficiency. Similarly to α‐AASA, 6‐oxo‐PIP not only accumulates in ALDH7A1 deficiency due to biallelic variants in *ALDH7A1,* but may also accumulate in secondary ALDH7A1 deficiency disorders, for example, in MoCD. However, our findings suggest analysis of urinary 6‐oxo‐PIP may not be a suitable biomarker for the diagnosis of ALDH7A1‐deficient neonates. Thus, a urine α‐AASA/P6C test remains more accurate. It remains unknown what impact the long‐term accumulation of 6‐oxo‐PIP would have on the brain and how it is affected by treatment. Recently, an *ALDH7A1* knockout mouse model was generated which shows similar biochemical changes to those seen in humans.^32^ This includes elevated α‐AASA, P6C and pipecolic acid in the brain and liver. This model could provide invaluable insight into the disease biochemistry via proteomic and metabolomic approaches, and potentially answer key questions on how 6‐oxo‐PIP synthesis is regulated.

## AUTHOR CONTRIBUTIONS

Conceptualisation: Youssef Khalil, Philippa B. Mills and Peter Clayton; Investigation: Youssef Khalil and Reddy Vootukuri; Methodology: Youssef Khalil and Matthew P. Wilson; Resources: Emma Footitt, Michael F. Wempe, Curtis R. Coughlin II, Spyros Batzios and Viktor Kožich. Supervision: Philippa B. Mills and Peter Clayton; Writing–original draft: Youssef Khalil; Writing–review and editing: Philippa B. Mills, Peter Clayton, Emma Footitt, Reddy Vootukuri, Matthew P. Wilson, Michael F. Wempe, Curtis R. Coughlin II, Spyros Batzios and Viktor Kožich. All authors approved the final manuscript as submitted.

## CONFLICT OF INTEREST STATEMENT

Youssef Khalil, Emma Footitt, Reddy Vootukuri, Spyros Batzios, Matthew P. Wilson, Viktor Kožich, Peter T. Clayton and Philippa B. Mills declare no conflicts of interest. Michael F. Wempe and Curtis R. Coughlin II are named as inventors for a patent describing 6‐oxo‐pipecolic acid quantitation by mass spectrometry (US‐20220057371‐A1).

## ETHICS STATEMENT

All procedures followed were in accordance with the ethical standards of the responsible committee on human experimentation (institutional and national) and with the Helsinki Declaration of 1975, as revised in 2000 (5). This article does not contain any studies with animal subjects performed by any of the authors. Informed consent was obtained from legal guardians for patients registered at Great Ormond Street Hospital NHS Trust (London‐Bloomsbury National Research Ethics Committee: REC reference: 13/LO/0168, IRAS project ID: 95005, Study of Inherited Metabolic Disease).

## INFORMED CONSENT

Informed consent was obtained from all patients for being included in the study.

## Supporting information


**Figure S1.** Supplementary Figure.

**Figure S2.** Supplementary Figure.

**Data S1.** Supporting Information.

## Data Availability

The data that support the findings of this study are available from the corresponding author upon reasonable request.
