# Supplementary figures and images for: Assessment of urinary 6‐oxo‐pipecolic acid as a biomarker for ALDH7A1 deficiency
Source: J Inherit Metab Dis. 2024 Jul 22;48(1):e12783. doi: 10.1002/jimd.12783 (PMC11670438; doi:10.1002/jimd.12783)

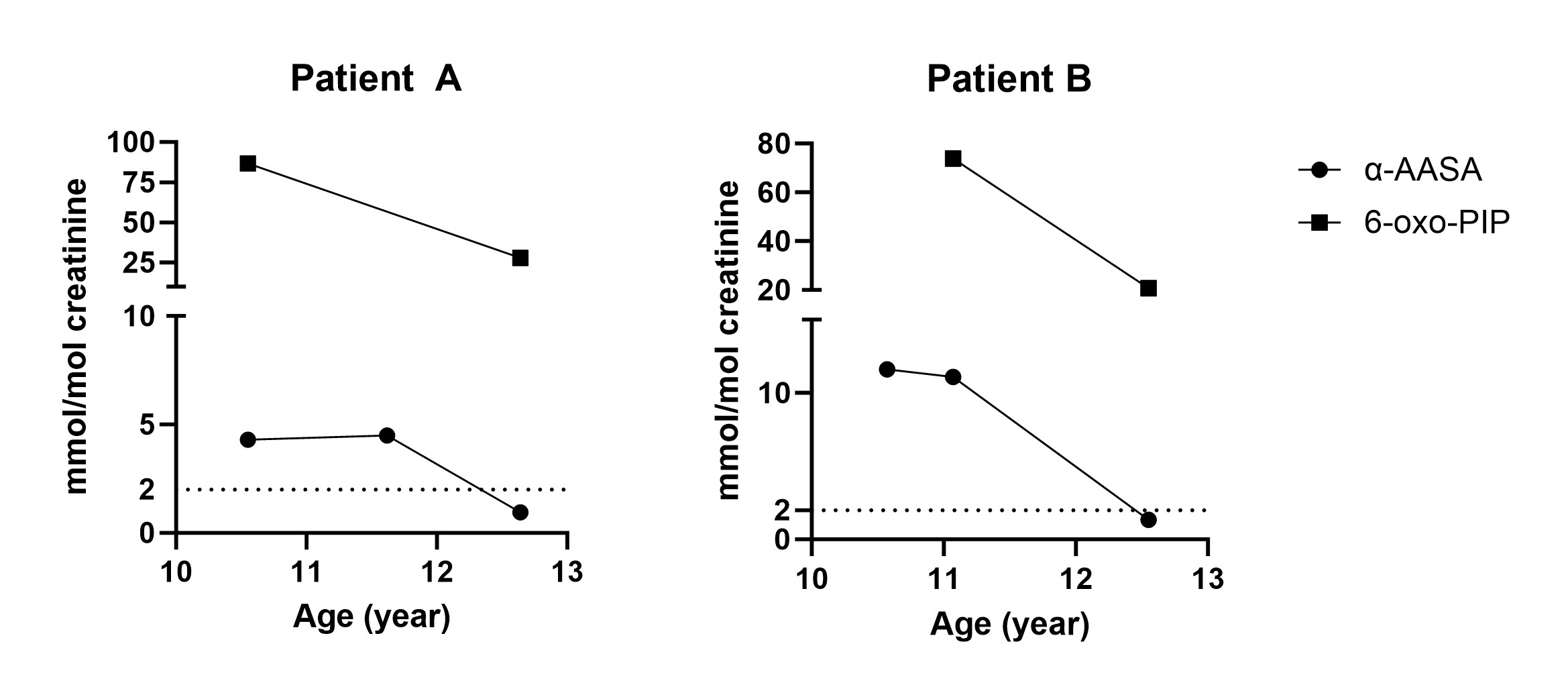

Supplement: Supplementary file 1 — Figure S1. Supplementary Figure. [file JIMD-48-0-s002.tif]

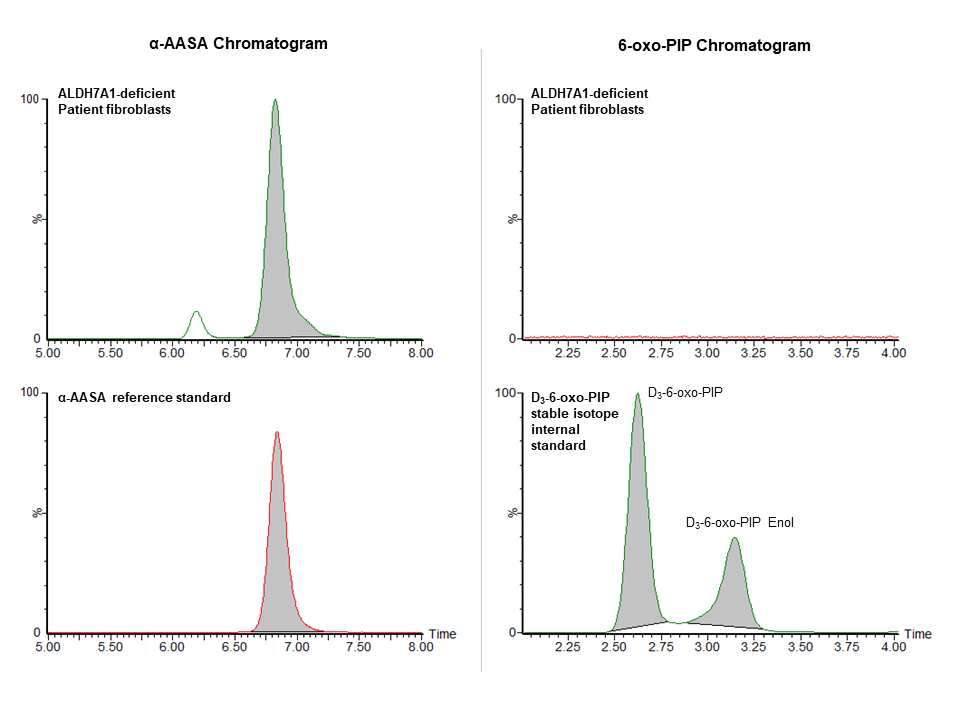

Supplement: Supplementary file 2 — Figure S2. Supplementary Figure. [file JIMD-48-0-s001.tif]
