# Supplementary material for: Assessment of urinary 6‐oxo‐pipecolic acid as a biomarker for ALDH7A1 deficiency
Source: J Inherit Metab Dis. 2024 Jul 22;48(1):e12783. doi: 10.1002/jimd.12783 (PMC11670438; doi:10.1002/jimd.12783)
Supplement: Supplementary file 3 — Data S1. Supporting Information. [file JIMD-48-0-s003.docx]

# Supplementary

Table S1. MRM transitions used for the detection of α-AASA and 6-oxo-PIP

|  | Precursor ion (m/z) | Product ion (m/z) | Cone voltage (V) | Collision Energy (V) | Ion Mode |
| --- | --- | --- | --- | --- | --- |
| α-AASA-FMOC | 366.1 | 144.1 | 4 | 20 | - |
| d_3_-AAA-FMOC | 385.02 | 163.06 | 19 | 13 | - |
| 6-oxo-PIP | 144.17 | 55.12 | 28 | 20 | + |
| 6-oxo-PIP | 144.17 | 70.15 | 28 | 18 | + |
| 6-oxo-PIP^1^ | 144.17 | 98.18 | 28 | 12 | + |
| D_3_-6-oxo-PIP | 147.17 | 58.12 | 28 | 20 | + |
| D_3_-6-oxo-PIP | 147.17 | 73.15 | 28 | 18 | + |
| D_3_-6-oxo-PIP^1^ | 147.17 | 101.18 | 28 | 12 | + |
| ^1^Quantitative | | | | | |

Figure S1. Longitudinal analysis of α-AASA and 6-oxo-PIP in two genetically confirmed ALDH7A1-deficient patients with normal urinary α-AASA but elevated 6-oxo-PIP levels. α-AASA control range above 12 months of age is below 2 mmol/mol creatinine.


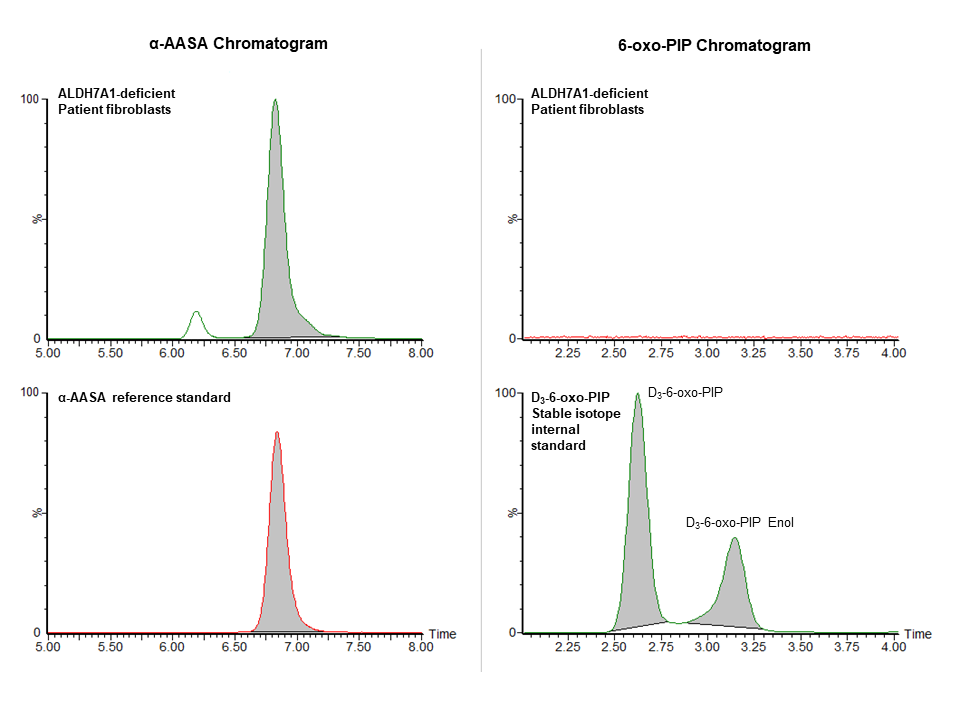


Figure S2. LC-MS chromatograms of α-AASA and 6-oxo-PIP extracted from ALDH7A1-deficient patient fibroblasts. α-AASA is detected in ALDH7A1-deficient fibroblasts cultured with lysine-rich media whilst 6-oxo-PIP is not detected.
